# Enhancement of mechanical strength and in vivo cytocompatibility of porous β-tricalcium phosphate ceramics by gelatin coating

**DOI:** 10.1186/s40729-016-0037-3

**Published:** 2016-02-06

**Authors:** Toshitake Furusawa, Tsutomu Minatoya, Toshimitsu Okudera, Yasuo Sakai, Tomohiro Sato, Yuta Matsushima, Hidero Unuma

**Affiliations:** 1 Graduate School of Science and Engineering, Yamagata University, 4-3-16 Jonan, Yonezawa, 992-8510 Japan; 2 Tohoku Oral Implant Association, 1-7-42 Hachihon-matsu, Sendai, 980-0001 Japan; 3 Kanagawa Dental College, 82 Inaoka, Yokosuka, 238-8580 Japan; 4 Tokyo Plastic Dental Society, 2-26-2 Oji, Kita-ku, Tokyo, 114-0002 Japan; 5 Jellice Co., Ltd., 4-4-1, Sakae, Tagajo, 985-0833 Japan; 6 Faculty of Engineering, Yamagata University, 4-3-16 Jonan, Yonezawa, 992-8510 Japan

**Keywords:** β-TCP, Gelatin, Compressive strength, Cytocompatibility

## Abstract

**Background:**

In an attempt to prepare scaffolds with porosity and compressive strength as high as possible, we prepared porous β-tricalcium phosphate (TCP) scaffolds and coated them with regenerative medicine-grade gelatin. The effects of the gelatin coating on the compressive strength and in vivo osteoblast compatibility were investigated.

**Methods:**

Porous β-TCP scaffolds were prepared and coated with up to 3 mass% gelatin, and then subjected to thermal cross-linking. The gelatin-coated and uncoated scaffolds were then subjected to compressive strength tests and implantation tests into bone defects of Wistar rats.

**Results:**

The compressive strength increased by one order of magnitude from 0.45 MPa for uncoated to 5.1 MPa for gelatin-coated scaffolds. The osteoblast density in the internal space of the scaffold increased by 40 % through gelatin coating.

**Conclusions:**

Coating porous bone graft materials with gelatin is a promising measure to enhance both mechanical strength and biomedical efficacy at the same time.

## Background

Porous hydroxyapatite (HA) and β-tricalcium phosphate (β-TCP) have long been clinically used for bone grafts because they enable perfusion of cells and other factors necessary for bone regeneration and because they allow bone ingrowth [1–3]. There have been a large number of works on the effect of porous structures on biological efficacy. For example, pores larger than 100 mm are essential for bone ingrowth into HA scaffolds [4, 5], and larger pores facilitate faster bone ingrowth [6]. Therefore, much effort has been devoted to the fabrication of scaffolds with pores as large as possible, and various processing techniques have been reported, although most of them were attempted on HA [7, 8]. Examples include freeze casting [8–10], sponge templating [11, 12], gel casting [13], particle templating [14, 15], whisker sintering [16], robocasting [17], extrusion deposition [18], and slurry foaming [19].

Although larger pores and porosities are favorable for faster bone ingrowth, they deteriorate the mechanical strength of the scaffolds. From a practical viewpoint, the compressive strength needs to be higher than approximately 1.0 MPa in order to avoid collapsing of the scaffolds during the handling for implantation. Therefore, there is a trade-off between the mechanical strength and the porosity. One of the promising methods to reinforce scaffolds without lowering its porosity or biomedical efficacy is to coat the scaffolds with biocompatible polymers because the infiltration of polymers into the microcracks of the scaffolds reduces the fracture origin [20]. The most widely used polymers are poly(lactic acid)- and poly(caprolactone)-based polymers [14–16, 21–25]. Others include glycerol sebacate [26], gelatin [14, 27], and collagen [28]. When scaffolds were coated with poly(lactic acid) or poly(caprolactone), the compressive strength generally increased [21–25]. However, the effects of those coatings on the biomedical efficacy of the scaffolds differ from report to report. In some cases, in vitro differentiation of MC3T3-E1 preosteoblast cells and bone marrow stroma cells were promoted [21], whereas in other cases, the initial attachment and proliferation were suppressed [16, 23, 26]. Biomedical evaluation was not quantitatively described in some reports [22, 25]. Some studies reported the in vitro cytocompatibility of collagen-coated scaffolds, in which the proliferation and differentiation of MG63 were enhanced [28] and the differentiation of rat-originated osteoblasts was promoted [29]. Still, in vivo evaluation of those polymer-coated scaffolds has been very scarce.

One of the present authors (SY) has developed endotoxin-free gelatin for regenerative medicine [30]. Gelatin is an inherently cytocompatible substance, and its mechanical strength can be enhanced by thermal cross-linking. Therefore, gelatin is a promising substance for the reinforcing coating of porous ceramic scaffolds.

In the present work, we first reinforced porous β-TCP scaffolds by gelatin coating, followed by thermal cross-linking. Then, the resultant scaffolds were evaluated for in vivo cytocompatibility from animal implantation tests.

## Methods

### Preparation of porous β-TCP blocks

Porous β-TCP scaffolds were prepared in our laboratory by sintering porous green bodies in the following manner. Commercial β-TCP powder (β-TCP −100, Taihei Chemical Industrial Co., Ltd., Osaka, Japan) was ground with an automatic agate mortar for 30 min to crush any coarse agglomeration. Then, 36.84 g of the β-TCP powder was added to the dispersion medium, which was prepared by dissolving 0.325 g of polyvinyl alcohol (polymerization degree 2000) and 3.0 g of an ammonium polyacrylate-based dispersant (Kaocera 2000, Kao Corp., Tokyo, Japan). The mixture was ball milled for 12 h. MgO (0.37 g) was added to suppress the phase transition during sintering, and the mixture was ball milled again for 1 h to prepare a well-dispersed slurry. The solid content of the slurry was approximately 45 vol%.

A foaming agent (6 mL, EMAL D-3-D, sodium polyoxyethylene alkyl ether sulfate, Kao Corp.) was added to 30 g of the slurry. The mixed slurry was then whisked with a kitchen blender. The whisked slurry was poured into a polymer mold approximately 40 × 40 × 50 mm in volume, frozen with liquid nitrogen vapor, and then lyophilized to give a porous green body. The green body was sintered at 1473 K for 12 h in ambient air to obtain β-TCP scaffolds. The porosity of the as-sintered scaffolds was 92 % as measured by the Archimedes method.

### Gelatin coating

Two kinds of gelatin were used: reagent-grade gelatin (Wako Pure Chemicals Ind., Ltd.) for the preliminary experiments and regenerative medicine-grade gelatin (RM-100, Jellice Co., Ltd.) for the final experiments. Porous β-TCP scaffolds were immersed in aqueous solutions containing 0.5, 1.0, or 2.0 mass% gelatin for 30 s, taken out, and the redundant solution was removed by wiping the blocks with paper towels. The β-TCP blocks bearing gelatin solutions were cooled in a refrigerator at 253 K overnight and then dried at room temperature in a vacuum. The dried, gelatin-coated β-TCP scaffolds were subjected to heat treatments in a vacuum to obtain cross-linked gelatin. The cross-linking temperatures were 373, 393, 413, and 433 K, and the duration was 12 h.

### Characterization of gelatin-coated β-TCP

The crystalline phase of the sintered scaffold was ascertained to be β-TCP by X-ray diffractometry (data not shown). The gelatin content was determined by thermogravimetry. The compressive strength of the β-TCP scaffolds, both gelatin-coated and uncoated, using samples approximately 10 × 10 × 20 mm in size was measured on an Aikoh testing machine at a crosshead rate of 1.0 mm/min. The compressive strength was defined as the maximum stress before the strain exceeded 10 % of the specimen length. The microstructure was observed with a scanning electron microscope (SEM, e-SEM, Shimadzu Rika Corp., Tokyo, Japan).

### Animal implantation test

The animal implantation tests were conducted under the permission of the Ethics Commission on the Animal Tests, Kanagawa Dental College (No. 2014-8.11-1). Male Wistar rats, 7 weeks of age, were used. A bone defect 5.2 mm in diameter was made in the cranial bone of each rat with a dental drill. Either a gelatin-coated or uncoated β-TCP block (samples E and O in Table 1) was implanted into the defect, and the skin was sutured. Each group contained nine rats. After 2 weeks, the experimental sections were retrieved, sliced into thin sections 3.5 μm in average thickness, decalcified, and stained with hematoxylin–eosin. One thin section was prepared from each rat. From each thin section, pictures from five fields of view were taken, and the number of osteoblasts in a 100 × 100 μm area in the internal space of the scaffold was counted. The statistical significance of the osteoblast density was examined by Student’s *t* test.

**Table 1:** Preparation conditions and physical properties of gelatin-β-TCP scaffolds

Sample	Gelatin content (mass%)	Cross-link temp (K)	Porosity (%)	Average compressive strength *σ* (MPa)	Standard deviation	Weibull coefficient
O	0.0	−	92	0.45	0.1	4.4
A	0.6	433	92	3.36	1.3	2.9
B	1.4	433	91	3.38	1.0	3.2
C	3.0	373	91	3.42	1.1	3.0
D	3.0	393	91	3.59	1.1	2.9
E	3.0	413	91	5.14	1.2	4.2
F	3.0	433	91	5.04	1.6	2.9

## Results

### Physical properties of the β-TCP scaffolds

Table 1 summarizes the preparation conditions and physical properties of the gelatin–β-TCP scaffolds. The contents of the gelatin coatings varied from 0.6 to 3.0 mass%, depending on the gelatin concentration in the coating solution. The decrease in porosity after the gelatin coating was small, and all the scaffolds had porosities higher than 90 %.

**Fig. 1:**
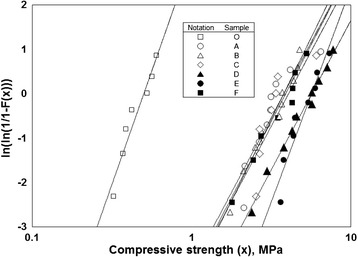
Weibull plots of the compressive strengths of uncoated and gelatin-coated scaffolds

**Fig. 2:**
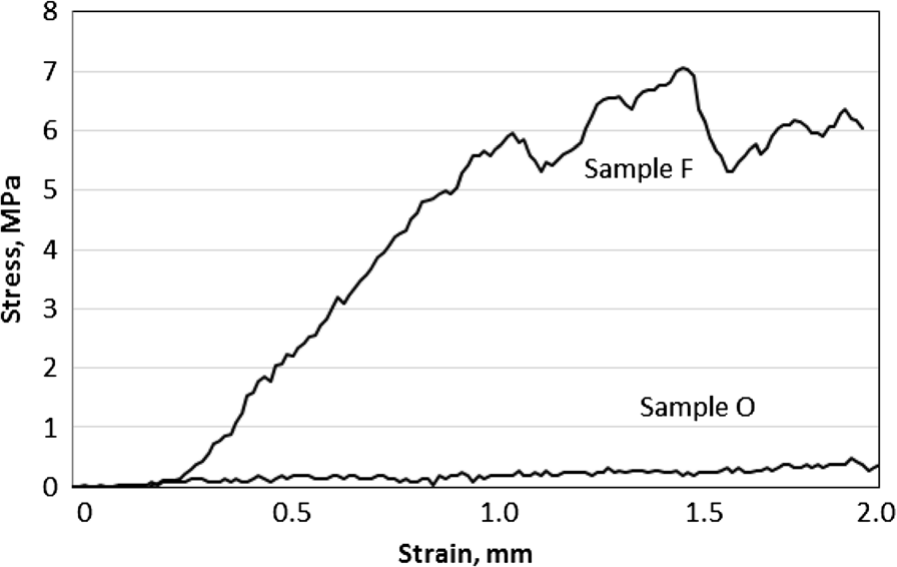
Examples of stress–strain curves of uncoated sample O and gelatin-coated sample F

A Weibull plot of the compressive strengths of the scaffolds is shown in Fig. 1, and the typical stress–strain curves of uncoated sample O and gelatin-coated sample F are shown in Fig. 2. The average compressive strength, standard deviation, and Weibull coefficient of each sample are given in Table 1. The enhancement of the compressive strength by the gelatin coating was remarkable; just 3.0 mass% of gelatin increased the compressive strength by one order of magnitude. The compressive strength increased with increasing gelatin content (comparing samples O, A, B, and F) and with increasing cross-linking temperature (comparing samples C, D, E, and F). Cross-linking, however, seemed to terminate at 413 K because there was no significant difference in the compressive strength between samples E and F. In spite of the increase in the compressive strength, the Weibull coefficient did not increase upon gelatin coating.

**Fig. 3:**
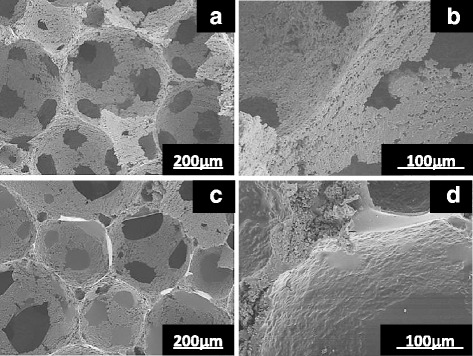
SEM pictures of **a**, **b** uncoated sample O and **c**, **d** gelatin-coated sample F

The microstructures of the uncoated and gelatin-coated scaffolds are shown in Fig. 3. The pore diameter seemed to be quite uniform, ranging from 200 to 500 μm. The gelatin layer was visible in the interconnections of the pores in the coated scaffold [Fig. 3c]. Under a higher magnification, infiltration of gelatin in the coated scaffolds was observed because pores smaller than a few micrometers were buried and the surface became smoother [Fig. 3d].

### In vivo tests

**Fig. 4:**
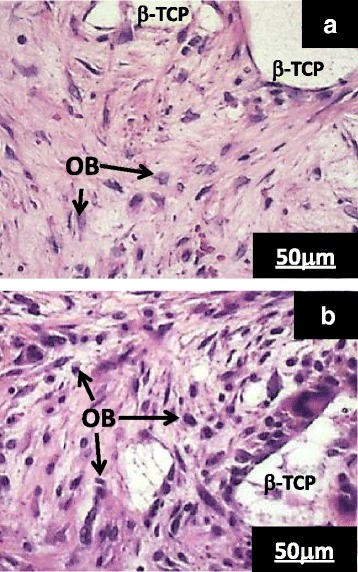
Histological pictures around implanted scaffolds: **a** uncoated sample O and **b** gelatin-coated sample E. β-TCP and OB stand for unresorbed β-TCP and osteoblast, respectively

**Fig. 5:**
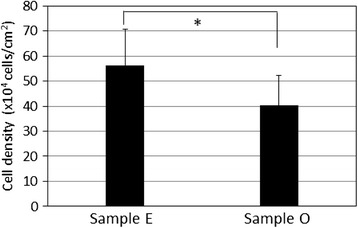
Osteoblast densities in the internal space of uncoated sample O and gelatin-coated sample E. *p < 0.05

Figure 4 shows histological photographs of the implanted gelatin-coated [Fig. 4a] and uncoated [Fig. 4b] scaffolds. In both pictures, white areas correspond to unresorbed β-TCP and blue dots correspond to osteoblasts. Newly formed bone was not yet recognized in either picture; however, the osteoblast density seemed to be higher near the gelatin-coated scaffold. The osteoblast density analysis is shown in Fig. 5. The osteoblast density around the gelatin-coated scaffolds (sample E) was higher than that around the uncoated scaffolds (sample O) by approximately 40 % with statistical significance: from 4 × 10^5^ cells/cm^3^ for the uncoated scaffolds (O) to 5.6 × 10^5^ cells/cm^2^ for the gelatin-coated scaffolds (E).

## Discussion

The present gelatin coating effectively reinforced porous β-TCP scaffolds. Generally, the fracture of brittle materials originates from the weakest crack tip where the applied stress is concentrated. The applied stress (*σ*) is concentrated at the crack tip to a value of *σ*_m_ depending on the depth (*c*) and curvature radius (*ρ*) of the crack tip, in the following manner:

1 $$ {\sigma}_m=2\sigma {\left(\raisebox{1ex}{$c$}\!\left/ \!\raisebox{-1ex}{$\rho $}\right.\right)}^{\raisebox{1ex}{$1$}\!\left/ \!\raisebox{-1ex}{$2$}\right.} $$

The fracture starts when *σ*_m_ exceeds the theoretical strength of the material, *σ*_th_. Therefore, the mechanical strength of a material increases as the cracks become less sharp and shallow. As shown in Fig. 3, the coated gelatin seemed to infiltrate into the microcracks of the framework of the scaffolds and flatten the framework surface, which should enhance the compressive strength of the scaffolds.

Presently, porous bone augmentation materials clinically used in Japan are fabricated so that the material possesses porosity as high as possible while retaining minimal compressive strength. To the authors’ knowledge, the lowest compressive strength of clinically used β-TCP scaffolds is 0.9 MPa (Osferion, Olympus Terumo Biomaterials, Tokyo, Japan) and its porosity is 75 %. In contrast, we have succeeded in preparing β-TCP scaffolds whose porosity and compressive strength are far higher than those of the commercial scaffolds. If the requisite minimum strength for bone augmentation material is approximately 1.0 MPa, there is a room to further increase the porosity.

In addition to reinforcing β-TCP scaffolds, the gelatin coating increased the osteoblast density near the scaffolds. This seems natural because gelatin has long been known to be a cytocompatible material. In the present study, however, 2 weeks of implantation may have been too short to observe the rates of the new bone formation within the pores of the scaffolds and of the resorption of those. Those studies will have to be conducted to more precisely and quantitatively assess the effect of gelatin coating. At least, still, a higher osteoblast density may imply faster new bone formation.

On the other hand, it has been well established that the dissolution of β-TCP promotes the migration of osteoclasts and osteoblasts [31], and calcium ions released from β-TCP may promote differentiation of osteoblasts [32, 33]. The gelatin coating may slow the dissolution of β-TCP, depending on the amount and thickness of the coating. Therefore, an increase in the osteoblast density alone does not guarantee fast bone regeneration. Although further studies are necessary to elucidate the biomedical efficacy of gelatin coating, this work is the first to report the in vivo effect of the gelatin coating on osteoblast density.

Hydrolysis of gelatin gives peptide oligomers. Among those, tripeptides, which consist of glycine and two other amino acids, have been proven to promote osteoblast differentiation [31] and in vivo bone healing [34, 35]. There is a possibility, therefore, that the coated gelatin is hydrolyzed to tripeptides, thus further promoting bone formation.

## Conclusions

Porous β-TCP scaffolds with approximately 90 % porosity were prepared and coated with gelatin. The gelatin coating and subsequent thermal cross-linking increased the compressive strength by one order of magnitude. The highest compressive strength attained was 5.1 MPa. The gelatin-coated and uncoated scaffolds were implanted into bone defects of the cranial bones of Wistar rats for 2 weeks. The osteoblast density in the internal space of the scaffold was enhanced by 40 % by gelatin coating, implying the possibility of faster bone formation.
